# Evaluation of Mussel Shells Powder as Reinforcement for PLA-Based Biocomposites

**DOI:** 10.3390/ijms21155364

**Published:** 2020-07-28

**Authors:** Vito Gigante, Patrizia Cinelli, Maria Cristina Righetti, Marco Sandroni, Leonardo Tognotti, Maurizia Seggiani, Andrea Lazzeri

**Affiliations:** 1 Department of Civil and Industrial Engineering, University of Pisa, 56122 Pisa, Italy; vito.gigante@dici.unipi.it (V.G.); info_lam@katamail.com (M.S.); leonardo.tognotti@unipi.it (L.T.); maurizia.seggiani@unipi.it (M.S.); andrea.lazzeri@unipi.it (A.L.); 2 National Research Council, Institute for Chemical and Physical Processes (CNR-IPCF), 56124 Pisa, Italy

**Keywords:** natural filler, calcium carbonate, thermal properties, mechanical properties, biocomposites

## Abstract

The use of biopolyesters, as polymeric matrices, and natural fillers derived from wastes or by-products of food production to achieve biocomposites is nowadays a reality. The present paper aims to valorize mussel shells, 95% made of calcium carbonate (CaCO_3_), converting them into high-value added products. The objective of this work was to verify if CaCO_3_, obtained from Mediterranean Sea mussel shells, can be used as filler for a compostable matrix made of Polylactic acid (PLA) and Poly(butylene adipate-*co*-terephthalate) (PBAT). Thermal, mechanical, morphological and physical properties of these biocomposites were evaluated, and the micromechanical mechanism controlling stiffness and strength was investigated by analytical predictive models. The performances of these biocomposites were comparable with those of biocomposites produced with standard calcium carbonate. Thus, the present study has proved that the utilization of a waste, such as mussel shell, can become a resource for biocomposites production, and can be an effective option for further industrial scale-up.

## 1. Introduction

Environmental concerns connected to financial needs as well as the implementation of smart policies, such as circular economy, bioeconomy etc. for the management of natural resources is leading to the development of new generations of materials. In this framework, the interest about polymer composites filled with natural-organic fillers is increasing [1,2]. Composites manufacturers are converting their production in order to improve and substitute traditional commodities with new ones with high content of biobased materials, recyclable or compostable, for sustainable and responsible actions.

The use of bio-polyesters and natural fillers, either organic or inorganic, for the production of biocomposites is nowadays already a reality in many sectors, ranging from agriculture and packaging to building construction components and other high value applications, in agreement with a bioeconomy approach [3,4].

Accordingly, research and industrial awareness is currently focusing on eco-friendly composites based on natural fillers from food production wastes due to low cost and large availability (e.g., shellfish, shrimp, oyster and mussel shells) [5]. These fillers constitute an effective alternative to conventional reinforcements, giving an added value to offcut by-products.

In this context shell wastes represent a substantial amount of by-products in the shellfish aquaculture. For an eco-friendly and economical disposal, it is highly desirable to convert these residues into high value-added products [6]. Bivalve mollusks represent almost 10% of the world’s total fishery production, while mussels represent 14% of mollusk production via aquaculture [7], and, of the entire amount of mussels produced, 75 to 90% consists of shells. These shells are composed of 95% calcium carbonate, and the remaining is organic matter such as glycoproteins, polysaccharides, glycosaminoglycan and chitin. Being the richest source of biogenic CaCO_3_, shell wastes are suitable to prepare high purity calcium carbonate powders, which have been extensively utilized as particle fillers in compounding and extrusion of polymer composites [8].

About biocomposites processing, CaCO_3_ cheapest grades are employed principally to reduce costs, favor mold release and promote disintegration in composites formulations [9]. The more expensive finest grades are used to increase elastic modulus and strength [10]. Processing aids, lubricants and anti-blocking additives like CaCO_3_ are commonly employed in flat die extrusions, because they are able to reduce the adhesion between films in the winding phase of blown film extrusion or calendering. Recently CaCO_3_ has been added also to polyprolylene (PP) in several ratios by using a twin screw extruder to improve PP wear characteristics [11].

Concerning CaCO_3_ produced from biogenic raw materials, Hamester et al. [8] used calcium carbonate from mussel and oyster shells as filler in polypropylene to improve the rigidity of the composite. Chong et al. [12] obtained fire-retardant plastic material from oyster-shell powder and recycled polyethylene. The seashell reinforcement in poly(methyl methacrylate) (PMMA) improved compressive strength and wear resistance [13]. Funabashi et al. [14] evaluated the presence of calcium carbonate from oyster shell powder in a blend of poly(butylene succinate) (PBS). According to Li et al. [15], the mechanical strength of PP composites with seashell carbonate was higher than that of traditional commercial calcium carbonate-filled PP, due to the formation of the β-crystal phase. An important feature is the polymorphic composition of calcium carbonate from mollusks, because the presence of the aragonite crystalline form is higher with respect to commercial calcium carbonate obtained from stones. The shape of the classical calcite crystals is generally cubic, whereas aragonite crystals are acicular, and when they are chopped, elongated particles can be obtained: this morphology certainly has a positive effect on the mechanical properties of the compound [16]. Another proof that biogenic calcium carbonate can improve the stiffness of a polymer was provided by Kunikate et al. [17], who, while investigating the strengthening mechanisms in calcite single crystals from mollusk shells, found that the plain strain indentation modulus of this crystal form can reach values of about 75 GPa.

The aim of the present work is to demonstrate that waste mussel shells provided from an aquaculture production in the northeast of Italy, can be valorized as fillers for polymeric matrices to produce sustainable biocomposites. The objective was to evaluate if the CaCO_3_ achieved from these mussel shells can guarantee an improvement of thermal, mechanical and physical properties to a compostable matrix based on polylactic acid (PLA) and poly(butylene adipate-*co*-terephthalate) (PBAT). The effect of an increasing content of mussel shell powder has been investigated from 5 to 20 wt%. PLA is one of the most used biopolymers, thanks to its large processability window, mechanical strength and high Young’s modulus [18]. However, it presents some drawbacks that must be overcome (low thermal resistance, brittleness, low toughness, slow crystallization rate) [19]. To achieve good values of flexibility and ductility, many attempts have been made and with various methodologies [20,21,22]. The addition of PBAT to PLA has been widely investigated in literature [23,24,25], also in different temperature conditions [26], and tailored formulations have been developed and tested in our research group for several applications [27]. In the present work PLA has been blended with a biodegradable elastomer, PBAT, in a weight ratio 3:1, as previously reported by the authors with a small amount of talc [28]. 

In order to possibly increase the nucleation rate in addition to calcium carbonate, a low weight percentage of talc has been added to the PLA/PBAT polymeric matrix. The use of inorganic particles as a nucleant for polymer crystallization is reported in the literature. Phetwarotai et al. [29] proved that tensile properties, thermal stability, spherulitic morphology and crystallization behavior of PLA blends significantly depend upon the weight percentage of the calcium carbonate and talc employed. A combination of inorganic fillers (talc and CaCO_3_ mixed together) may led to a significant improvement of the nucleation, according to Leong et al. [30]. The same synergistic effect was also evaluated by Shi et al. [31], who demonstrated that the half crystallization time of pure PLA is reduced by the addition of talc.

This work aims to evaluate the mechanical, morphological and thermal effects of mussel shell powder waste in extruded PLA/PBAT biocomposites. The method to achieve a tailored distribution of shell powder dimensions is discussed and an analytical investigation on the adhesion and compatibility between the polymeric matrix and the fillers is presented. This study leads to potentially compostable formulations than can be proposed for several practical applications. Indeed, according to a European directive (EN13432) [32], a material, in order to be defined as compostable, must mineralize by 90% within 6 months. Biodegradation is a slow process carried out by nature, and if this biological process is complete, there is a total conversion of the starting organic material into simple molecules, such as water, carbon dioxide, or methane if in anaerobic conditions [33]. Biocomposites based on compostable ingredients are considered compostable if able to disintegrate under 2 mm in three months in composting plants. The presence of fillers, even inorganic, is known to promote materials’ disintegration. 

The present paper fits into the stream of a circular economy approach concepts to be practiced in all sectors. The use of a problematic waste that can become an added-value product in a field such as biocomposites production could also be a viable opportunity for a further industrial scale-up.

## 2. Materials and Methods

### 2.1. Materials and Biocomposites Mass Compositions

The materials used for this work were:

- PLA2003D purchased from NatureWorks (thermoforming and extrusion grade), with melt flow index (MFI): 6 g/10 min (210 °C, 2.16 kg), nominal average molar mass: 180,000 g/mol, density: 1.24 g/cm^3^. It contains about 4% of D-lactic acid units, which lower the melting point and the crystallization tendency, improving the processability during the melting extrusion.
- PBAT: Ecoflex^®^ C1200 purchased from BASF, is a biodegradable, random aliphatic-aromatic copolyester based on the monomers 1.4-butanediol, adipic acid and terephthalic acid with melt flow index (MFI): 2.7–5 g/10 min (190 °C, 2.16 kg), nominal average molar mass: 126,000 g/mol, density 1.26 g/cm^3^.
- Jetfine^®^ 0.7CA, supplied by Imerys, was the Talc employed in the formulations, with a median diameter of 0.7 μm, which results in very low particle sizes compared to conventional talc, extremely pure with ultra-lamellarity aspects that provide outstanding stiffness/impact strength balance in polymers. This talc grade imparts superior scratch and mar resistance and improves nucleation.
- The mussel shells used in this experimental research (Mytilus Edulis, also called blue mussels) were obtained from the Mediterranean Sea, washed with hot soapy water, calcinated at 200 °C for 1 h, dried and crushed into smaller pieces manually. Thereafter, the shell powder was obtained by means of a rotating blade lab blender and sieved to achieve a micrometric filler.

In Table 1 the weight composition of the extruded and tested formulations is reported. As described in the processing section, filler particulate was added (with increasing concentration) to a polymeric matrix of PLA/PBAT, with the weight ratio maintained at 3:1, in order to obtain a morphology characterized by an elastomeric phase dispersed in a more rigid continuous phase. The blend PLA/PBAT with weight ratio 3:1 was studied in a previous work of the same research group [27], and a considerable improvement from the point of view of elongation at break and Charpy Impact characteristics with respect to pure PLA was evidenced. Despite the ductility increase, stiffness was found not to decrease significantly. Also the viscosity values are compatible with the use in processing such as injection molding.

### 2.2. Processing and Testing Methodologies 

The mussel shells, after washing and grinding as described in the previous paragraph, were dried in a ventilated electric oven at a temperature of 110 °C for 24 h before being processed. Polymers, instead, were maintained in a DP 604-615 dryer at 50 °C (Piovan S.p.A., Venice, Italy) before the extrusion. The biocomposites were produced by means of a single screw extruder (BRABENDER Plasticorder KE 19, Duisburg, Germany) with a screw diameter of 19 mm and a ratio L/D = 40, equipped with single hole die head (2.5 mm) and a small water-cooling bath, working with material amounts from 0.2 to 1.5 kg. This lab extruder can be controlled remotely by a PC interface managed by a proprietary software in both the operational and the data acquisition functionality. By virtue of its small dimension, it resulted optimal in the preparation of small thermoplastic specimens. The temperature profile adopted in extrusion was the following: 170 |175|180 °C. At the end of the extrusion process, the obtained strands were granulated in a COMAC pelletizer (Milan, Italy) with rotating blades and the granules were dried in the Piovan dryer at 55 °C for more than 24 h before the successive operations. During the extrusion the distribution of temperature between the head and the rear of the extruder barrel was controlled to avoid mechanical screw overloading by reducing the extrusion pressure, and to prevent the overheating of the rear zone, in order to ensure a good and progressive gas discharge from the feeding hole, because no intermediate venting aperture was present along the barrel. The screw rate was settled up and maintained at its optimal value during the entire extrusion (from 40 to 60 rpm with a stable screw torque of 50 ± 5 Nm) achieving a feeding flow rate of 50 g/min. No anomalous torque variation nor any considerable change was observed in the extruded filament appearance during the operation.

Injection molding process was achieved through a Thermo Scientific HAAKE Minijet II (injection temperature = 180 °C, molding temperature = 60 °C, molding time = 15 s, injection pressure = 700 bar). Haake type III dog-bone specimens (size: 25 × 5 × 1.5 mm) for tensile tests and ISO 179 parallelepiped samples (size: 80 × 10 × 4 mm) for Charpy Impact were prepared. Before thermal and mechanical tests, the specimens were stored in a climate chamber at room temperature and controlled relative humidity of 50%.

Differential Scanning Calorimetry (DSC) measurements were performed with a Pyris (Perkin Elmer Instrument, Waltham, MA, USA) equipped with a Perkin Elmer IntraCooler 1 as a refrigerating system, which is a subambient device that automatically control the flow of a coolant through a circulating chamber attached to the Pyris. The instrument was calibrated in temperature and enthalpy with high purity standards, according to the procedure for standard DSC, Dry nitrogen was used as purge gas at a rate of 25 mL/min. Samples of about 10 mg were analyzed from −10 °C to 200 °C at the heating rate of 10 °C/min, with an aluminum empty pan as reference.

Tensile tests were carried out at room temperature and at a crosshead speed of 10 mm/min by an Instron 5500 R universal testing machine (Canton, MA, USA) equipped with a 10 KN load cell and interfaced with a computer running MERLIN software (version 4.42S/N-014733H). At least five specimens for each composite were tested and the average values were reported.

Impact tests were performed at room temperature on ISO 179 V-notched specimens (V-notch 2 mm at 45°) using a 15 J Charpy pendulum of an Instron CEAST 9050 (Canton, MA, USA). The standard method ISO179:2000 was followed, operating at room temperature. For each blend, at least five specimens were tested.

In order to investigate the microstructure, the length and diameter distribution of grinded mussel shells, and the matrix/filler adhesion, the shells powder and the cryofractured biocomposites were analysed by Scanning Electron Microscope (SEM) FEI Quanta 450 FEG (ThermoFisher, Waltham, MA, USA) equipped with a Large Field Detector for low kV imaging simultaneous secondary electron (SE). The samples were previously gold sputtering by using a sputter coater Edward S150B.

Thermogravimetric analysis (TGA) was performed on mussel shells residues in form of powder using a TA Q-500 (TA Instruments, Waters LLC, New Castle, DE, USA). About 15 mg of sample into a platinum pan and heated from room temperature to 700 °C at 10 °C/min under nitrogen atmosphere. TGA was used to evaluate the thermal stability of mussel shells in view of its processing by melting extrusion.

## 3. Results and Discussions

### 3.1. Thermogravimetrical Analysis

**Figure 1:**
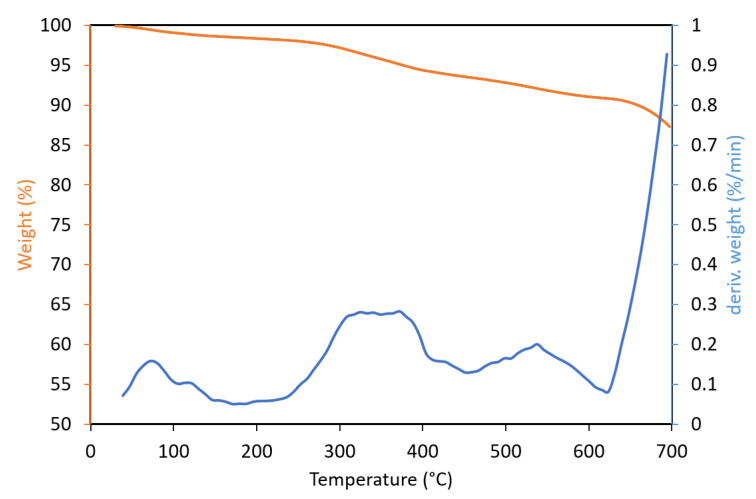
Thermogravimetric analysis of mussel shell residue.

TGA analysis (Figure 1) displayed that up to 200° C, the weight loss was about 2% due to the release of moisture residue. A further increase in temperature, from 300 to 400 °C, resulted in a weight loss of additional 3%, probably as consequence of oxidation and removal of volatile matter. Drastic weight loss, connected to decomposition of the mussel shells, appeared over 600 °C. This is corroborated by the observation 

This finding, which is corroborated by the observations of Mo et al. [34] and Rahmani et al. [35], indicates that calcium carbonate from mussel shell wastes can be used as a filler for many thermoplastic materials, which are generally processed below 400 °C.

### 3.2. Morphology Analysis and Mussel Shells Microstructure 

**Figure 2:**
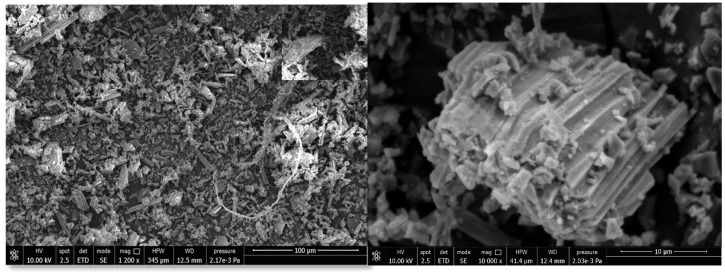
Micrographs at 1200× and 10,000× of shell powder.

In order to better understand the thermal and mechanical properties of the PLA/PBAT biocomposites with mussel shells, the granulometry distribution of the milled and sieved filler was evaluated. More than 400 particles were examined from five micrographs acquired by SEM analysis (Figure 2) by using the ImageJ^®^ software, to evaluate the length and diameter distribution of shell powder. 

The microstructure of mollusk shells has been reported in detail by Martinez-Garcia et al. [7]. According to their work prismatic layer rich in CaCO_3_ was observed in the SEM figures. Lertwattanaruk et al. [36] also observed that in mollusk shell powder (clam, mussel, oyster and cockle shells), the morphology showed irregularly shaped particles and multi-angle shapes.

**Figure 3:**
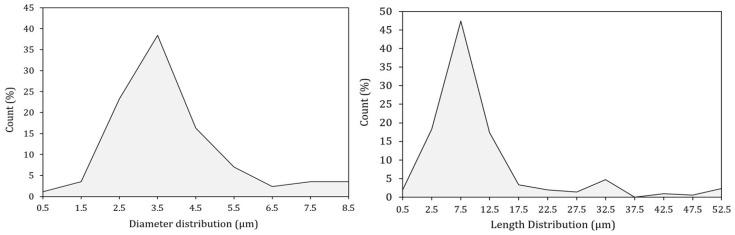
Dimensional analysis of shell powder.

The aspect ratio of the filler studied in the present work was estimated through the ratio between the weighted average of the lengths and the diameters. Analyzing the distribution shown in Figure 3, more than 88% of the particles are between 1 and 15 μm long and approximately 85% of them have a diameter between 2 and 6 μm. These average dimensions are well highlighted by the magnification at 10,000× in Figure 2.

This high concentration of fine particles and the minor presence of larger particles lead to a more heterogeneous distribution with respect to the commercial CaCO_3_. From the dimensional analysis it follows, therefore, that the filler used in this work exhibits an average weight aspect ratio of 2.6.

### 3.3. Thermal Analysis 

The heat flow rate curves of the as prepared biocomposites, measured at 10 K/min from −10 to 200 °C, after rapid cooling from *T_room_* to −10 °C, are shown in Figure 3. Due to the immiscibility of PLA and PBAT [24], and the minor percentage of PBAT in the polymeric matrix, only the thermal events involving PLA have been investigated. The glass trandition of PBAT is located at a very low temperature, around −30 °C, and its crystallinity is suppressed when blended with PLA.

The glass transition of PLA is observed in proximity of 60 °C for all the samples, in agreement with literature data and the immiscibility of the PLA/PBAT blend [37]. Before the melting endotherm, all the curves display an intense cold crystallization peak, which shifts to progressively lower temperatures with increasing the shell powder percentage, which attests the nucleating action exerted by the fillers on the PLA cold crystallization. 

During the cold crystallization process, α′-crystals mainly grow at temperatures lower than 100 °C, whereas a mixture of α′- and α-crystal develop in the temperature range from 100 and 120 °C [38]. Reorganization and recrystallization events overlap the entire fusion process. At a heating rate of 10 K/min, the disordered α′-form transforms into the more ordered α-phase via melting and almost simultaneous recrystallization [39], with the result that the fusion of the α′-crystals is not visible, being masked by the concomitant recrystallization, and the double melting behavior that extends from approximately 130 to 160 °C, has to be connected to the fusion of original α-crystals, grown during cold crystallization, and, at higher temperatures, α-crystals recrystallized upon heating [40,41]. This interpretation of the melting behavior is confirmed by the progressively reduced area of the first melting peak centered at about 148 °C, which parallels the shift of the cold crystallization to lower temperatures, with the consequent growth of a lower percentage of original α-crystals.

**Figure 4:**
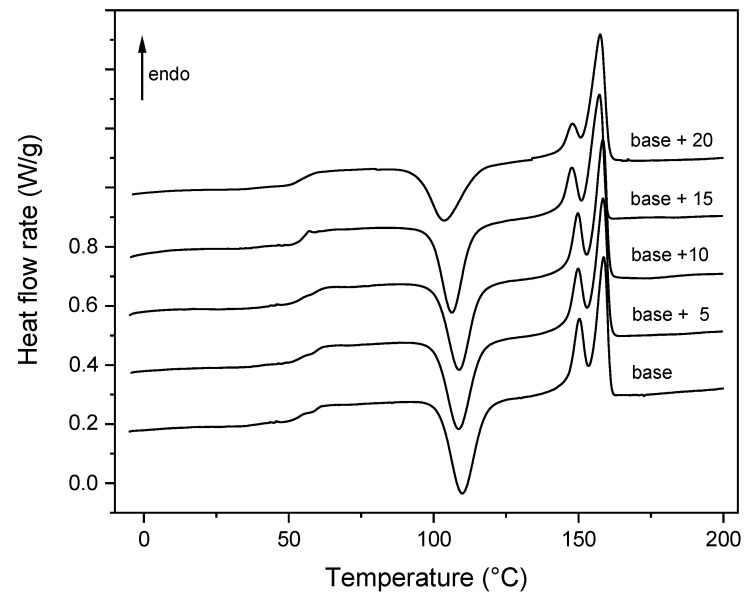
Heat flow rate curves of the PLA/PBAT matrix (base) and the biocomposites with increasing mussel shells content, as a function of the temperature. The ordinate values refer only to the bottom curve. All the other curves are shifted vertically for the sake of clearness.

Table 2 lists the enthalpy of cold crystallization (Δ*h_c_*) and the enthalpy of fusion (Δ*h_m_*) of the PLA/PBAT matrix and biocomposites, calculated from the heat flow rate curves shown in Figure 4. The Δ*h_c_* and Δ*h_m_* values collected in Table 2 are normalized to the PLA content. An estimation of the crystalline weight fraction that grows during the cold crystallization process (*w_Cc_*) and disappears during the melting process (*w_Cm_*) was obtained by dividing Δ*h_c_* and Δ*h_m_* by the enthalpy of the fusion of 100% crystalline PLA (Δ*h_m_*°) at the crystallization and melting temperatures, respectively, because Δ*h_m_*° increases with temperature [42]. As both α′- and α-crystals grow during cold crystallization, and disappear during the melting process, average values between the enthalpy of fusion of the α′- and α-forms were utilized. Thus, Δ*h_m_*° = 100 J/g for the cold crystallization and Δ*h_m_*° = 121 J/g for the melting process were utilized [42]. The *w_Cc_* and *w_Cm_* values listed in Table 2 reveal that the matrix and the biocomposites were, within the experimental error, amorphous after molding at 60 °C.

### 3.4. Tensile Results 

**Figure 5:**
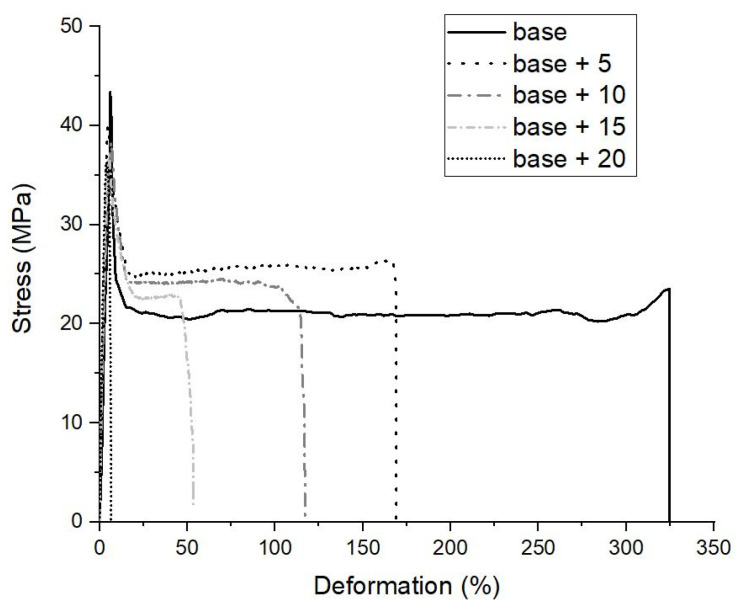
Stress-strain curves of the biocomposites.

Tensile test analysis was used to evaluate the possible utilization of mussel shells from food industry as a filler for polymeric formulations (Figure 5).

The incremental addition of calcium carbonate in the form of mussel shells powder leads to a stiffening of the biocomposites, i.e., an increase in the elastic modulus, and a slight decrease in the maximum stress (Table 3). Since the modulus of inorganic particles is usually much higher than that of the polymer, generally the elastic modulus of a composite is higher than that of the matrix. The PLA/PBAT biocomposites with mussel shells confirm this expectation, considering that the plain strain indentation modulus of calcite single crystals from mollusk shells can reach values of about 75 GPa [17].

The strength of a composite is a function of the weakest fracture path throughout the material. Hard fillers such as calcium carbonate can affect the strength in two ways: with a weakening effect, if they act as stress concentrators, or with a reinforcing effect, if they serve as barrier to crack growth [43]. The present biocomposites are part of the first case, although the decrease in the strength with respect to the matrix is small. Several factors significantly affect the mechanical properties and performance of biocomposites. The main disadvantage of natural fillers is their frequent incompatibility with the polymeric matrix, which results in poor adhesion and consequent reduction in tensile strength. This occurrence is also reflected in the decrease in the elongation at break increasing the content of CaCO_3_. It can be noted, indeed, that the deviation with respect to the PLA/PBAT matrix is high as far as the elongation at break is concerned, because during the uniaxial tensile test, shell particles act as stress intensification factors, causing the breakage of the small Haake Type 3 specimens.

The flexibility, in fact, is provided by the introduction of PBAT into the matrix; this property is progressively reduced by the addition of mussel shell, but only with 20 wt% of shells there was a transition from a ductile breakage (with consequent necking and plastic deformation of the dog-bone specimens) to fragile behavior (Figure 5). 

In ductile matrices such as the blend PLA/PBAT used in this work, particulate fillers cause an embrittlement of composites if there is poor interfacial adhesion. Micro-cavitation and micro-debonding can occur, introducing cracks of significant sizes [44]. Calcium carbonate can give rise to an improvement in fracture toughness when the mechanism of debonding is triggered in the presence of brittle matrices [45]. *Charpy impact strength values test*, performed on the PLA/PBAT biocomposites with mussel shell powder (see the last column of Table 3) revealed that the energy absorbed was approximately half with respect to that of the ductile matrix. The values obtained for the formulations with 10 and 15 wt% of shells were found higher than that of pure PLA and other biodegradable biocomposites obtained from food processing waste [46,47]. 

In particular, the Biocomposite with 15 wt% of mussel shell represents the optimum from the point of view of impact resistance. Evidently, mussel shell addition up to 15 wt% guarantees an obstacle to the propagation of the cracks by increasing the energy absorbed before the breakage, as also occurs for other biocomposites [48,49,50]. Over 15 wt%, the mussel shell content becomes excessive and acts as stress concentrator.

These results prove that the PLA/PBAT biocomposites with mussel shell are materials suitable for applications where moderate impact resistance is required.

In conclusion, the biocomposite with the intermediate content of mussel shells (10 wt%) showed an elastic modulus of 2.35 GPa, maximum stress of about 38 MPa, Charpy Impact Strength around 5 kJ/m^2^ and an elongation at break of more than 100%, which makes it usable for the production by injection molding of issues and objects, when a certain flexibility as well as stiffness are required. A deeper examination of the mechanical properties trend for the PLA/PBAT biocomposites with shell powder, and an analysis of the matrix/filler adhesion, is reported in the next paragraph.

#### Predictive Models Applied to Tensile Results

To explain the experimental tensile data and correlate them to the filler and matrix characteristics, predictive models have been applied, to better understand the reinforcement micromechanics mechanisms.

**Figure 6:**
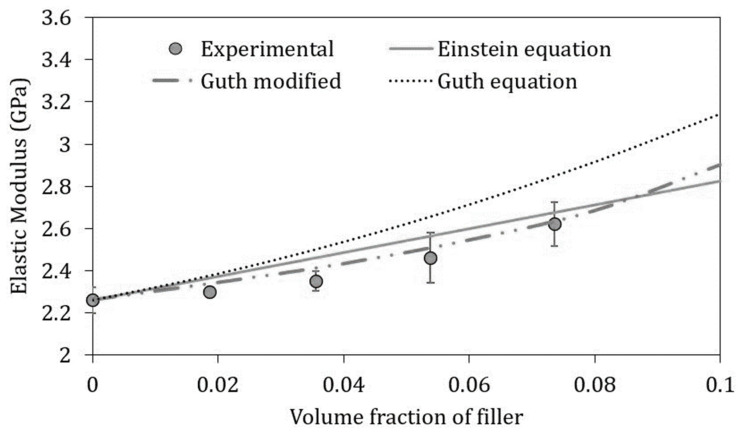
Comparison between the experimental elastic moduli of the PLA/PBAT biocomposites with mussel shells and the values predicted according to the different equations reported in the legend.

Many empirical equations have been proposed to predict the modulus of particulate–polymer as a function of the filler volume fraction. The modulus of inorganic particles is generally much higher than that of the polymer matrices; therefore, an increase in the elastic modulus is expected for composites. Einstein’s equation [51], one of the first equations used to predict the elastic modulus of composites containing rigid particles, which is generally applied at low particle loading, assumes that the composite modulus is independent of particle size, and predicts a linear relationship between the elastic modulus of the composite (*E_c_*) and the volume fraction of the particles (*V_p_*) (Equation (1)):

(1) $$E_{c}=E_{m}\left(1+2.5V_{p}\right)$$

where *E_m_* is the elastic modulus of the matrix. Figure 6 shows that the linear trend predicted by Equation (1) does not fit experimental data. Thus, the equation proposed by Guth [52], which adds a particle interaction term in the Einstein’s equation, was utilized:

(2) $$E_{c}=E_{m}\left(1+2.5V_{p}+14.1V_{p}^{2}\right)$$

The trend predicted by Equation (2) is not satisfactory, because the increase in the elastic modulus as a function of the filler amount appears overestimated.

The cause has to be found in the not perfect sphericity of the mussel shell residue. These results suggested to modify the Guth equation by considering the shape of the mussel shells particles, which is ellipsoidal [53]. This modification is here proposed (Equation (3)) by taking into account the mussel shells powder aspect ratio (*a_r_*):

(3) $$E_{c}=E_{m}\left[1+0.67\cdot V_{p}\cdot a_{r}+1.62\cdot V_{p}^{2}\cdot a_{r}^{2}\;\;\right]$$

The imperfect sphericity of the filler negatively influences the increase in stiffness especially at low values of volumetric fraction of mussel shell residue, resulting in values below the linearity predicted by Einstein’s equation. However, by increasing the CaCO_3_ content, the increase in elastic modulus returns to being exponential. For this reason, Guth also proposed Equation (3), to take into account the lack of stiffness increase for small quantities of filler that are not perfectly spherical.

As shown in Figure 6, this predictive model correctly fits the experimental data, predicting a small increase at low filler content, and an exponential increase when the interaction effect of the non-spherical particles becomes significant.

**Figure 7:**
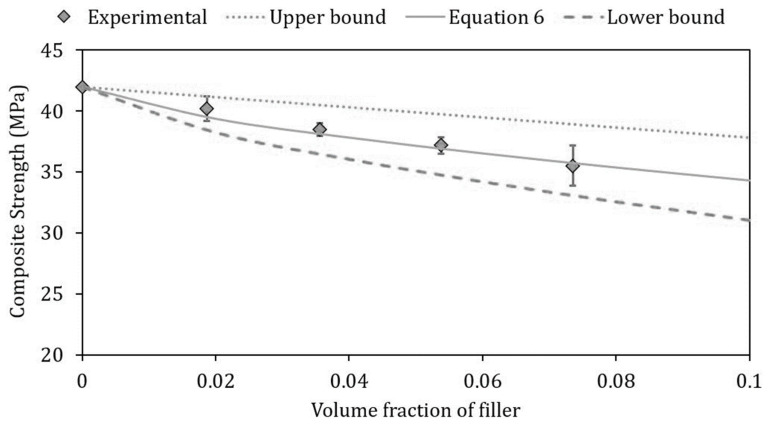
Comparison between the experimental strength of the PLA/PBAT biocomposites with mussel shells and the values predicted according to the different equations reported in the legend.

Concerning the composite strength behavior, factors such as particle size, particle/matrix interfacial strength and particle loading significantly affect the stress transfer between the matrix and the fillers [54]. The slight decrease in the maximum strength with the filler concentration (see Table 3) can be due to an absence of stress transfer from the matrix to the particles or to negligible stress field around the rigid particle. If there is no adhesion between the fillers and the polymeric matrix, and the load is sustained just by the matrix, a predictive model based on simple geometric considerations was proposed [55]:

(4) $$\sigma_{c}=\sigma_{m}\left(1-1.21V_{p}^{2/3}\right)$$

where σ_c_ and σ_m_ are the strength of the composite and the matrix, respectively. The calculated σσ_c_ values represent the lower bound of Figure 7. Conversely, if the strength of a particulate filled polymer composite is determined from the effective sectional area of load-bearing matrix in the absence of the particles, a very simple expression for the composite strength is given by Equation (5) [56], which represents the upper bound of Figure 7.

(5) $$\sigma_{c}=\sigma_{m}\left(1-V_{p}\right)$$

As shown in Figure 7, the experimental data are precisely within the range represented by Equations (4) and (5). An accurate fitting appears to be given by the predictive model of Nicolais and Nicodemo [57] (Equation (6)) in which the decrease in the composite strength with the increase of the filler content is obtained multiplying the filler volume by a factor 0.85, which presumes minimum adhesion and a small stress transfer between matrix and particles:

(6) $$\sigma_{c}=\sigma_{m}\left(1-0.85V_{p}^{2/3}\right)$$

**Figure 8:**
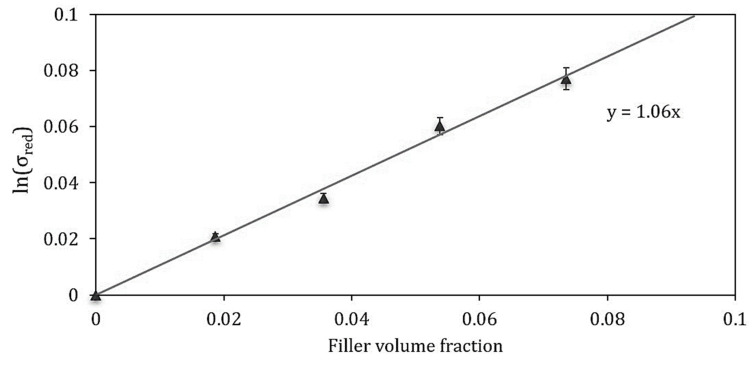
Reduced tensile strength as a function of the filler volume fraction for the determination of Pukanszky’s B parameter for the developed biocomposites with mussel shells powder.

To predict the strength behavior, which is generally strongly affected by the adhesion between the particles and the matrix, much more than stiffness, an additional model was applied to the PLA/PBA biocomposites under investigation. To verify if the adhesion between the PLA/PBAT matrix and the mussel shells is not zero, the Pukánszky’s model [58], one of the most used approach for composites with short fibers, was utilized. In this analytical model, the reinforcing effect of a filler is expressed quantitatively by considering the effect of the decrease in effective load-bearing cross-section of the polymer (Equation (7)):

(7) $$\ln\sigma_{c,red}=\ln\frac{\sigma_{c}\left(1+2.5\;V_{p}\right)}{1-V_{p}}=\ln\sigma_{m}+BV_{p}$$

where σ*_c_*_,*red*_ is the reduced tensile strength, i.e., the tensile strength normalized to the cross-section perpendicular to the load direction, *σ_c_* and *σ_m_* are the stress at break of the composite and the matrix, *V_p_* is the filler volume fraction, and *B* is a parameter connected to the matrix/filler interaction. From the slope of the logarithm of the reduced stress against the filler volume content, the value of the *B* parameter can be derived. *B* has no direct physical meaning, but it is connected with the interfacial properties of the system. For the PLA/PBAT biocomposites with mussel shells, the *B* parameter is around 1, as shown in Figure 8, which demonstrates that a weak interfacial bonding exists from matrix and filler. Interfacial interactions between the PLA/PBAT matrix and the shells particles favor only a small decrease in the composite strength with increasing the filler percentage.

### 3.5. Microscopic Interfacial Interaction

**Figure 9:**
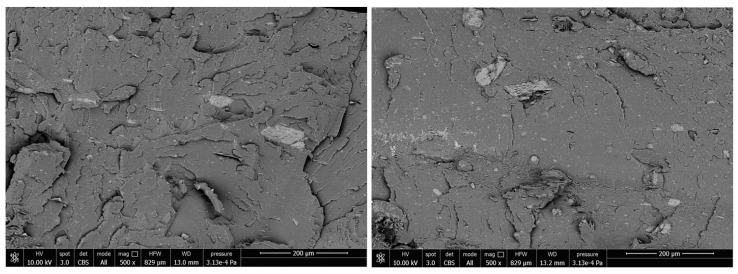
Back Scattering Images of base + 10 (**Left**) and base + 20 (**Right**) biocomposites.

To confirm the mechanical properties described in Section 3.4, microscopic interfacial interaction between the matrix and the fillers has been investigated through SEM analysis of cryo-fractured surfaces of base + 10 and base + 20 samples. In Figure 9, back scattering images illustrate the homogeneous distribution of particles with the low aspect ratio filler in both the biocomposites. More evident, but in a smaller number, are the particles with length in the order of 50 microns.

**Figure 10:**
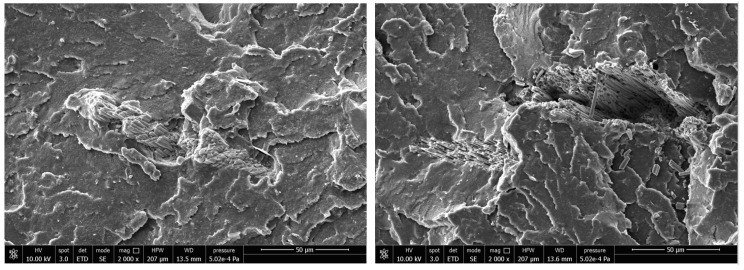
2000× magnification of interaction matrix/filler for base + 10 (**Left**) and base + 20 (**Right**) biocomposites.

Weak adhesion between the shell and the surrounding matrix, characterized by the presence of cracks and high porosity in the interfacial zone, is evidenced by the 2000× magnification (Figure 10), which confirm the results of the predictive models.

## 4. Conclusions

In this work, CaCO_3_ obtained from Mediterranean Sea mussel shells was used to prepare biocomposites with a polymeric matrix based on PLA and PBAT with a weight ratio of 3:1. The effect of an increasing content of mussel shell powder, from 5 to 20 wt%, was investigated. All the biocomposites turned out to be completely amorphous, due to the low molding temperature and time adopted for their processing.

Regarding the mechanical properties, a slight increase in the elastic modulus and a small decrease in the break strength was detected. The increase in the elastic modulus was due to the much higher modulus of the inorganic fillers with respect to the polymeric matrix. The small decrease in the strength has been connected to a weakening effect exerted by the mussel shell particles, which can act as stress concentrators. However, a small interfacial adhesion was present between the PLA/PBAT matrix and the fillers, because the strength decrease was found to be minor. The biocomposite with the intermediate content of mussel shells of 10 wt% showed an elastic modulus of 2.35 GPa, maximum stress of about 38 MPa, Charpy Impact Strength around 5 kJ/m^2^ and an elongation at break of more than 100%, which makes it usable for the production by injection molding of items and objects, when a certain flexibility as well as stiffness are required.

Moreover, to correlate mechanical experimental results with the filler and matrix properties, predictive models were applied, also to better understand the reinforcement micromechanics mechanisms. The elastic modulus trend was correctly predicted by considering the average aspect ratio of the mussel shells powder, whereas from the strength data, a parameter connected to the matrix/filler interaction was derived.

In conclusion, the use of a waste as mussel shells has allowed obtaining biocomposites with satisfactory mechanical properties, improving the elastic modulus, without significantly lowering the composite strength and the Charpy Impact strength, despite the weak adhesion/interaction between the matrix/particles. These biocomposites could become a viable solution also for a further industrial scale-up. However, to improve the matrix-filler interfacial bonding, a filler modification could be useful. Further efforts are thus necessary to develop biocomposite materials with improved mechanical properties for a wider range of applications.

## Figures and Tables

**Table 1:** Composition of the biocomposites.

Sample Name	PLA (wt%)	PBAT (wt%)	Talc (wt%)	Shells (wt%)
base	73.5	24.5	2	0
base + 5	69.5	23.5	2	5
base + 10	65.7	22.3	2	10
base + 15	62.0	21.0	2	15
base + 20	58.3	19.7	2	20

**Table 2:** Enthalpy of cold crystallization (Δ*h_c_*) and enthalpy of fusion (Δ*h_m_*), crystalline weight fraction growing during cold crystallization (*w_Cc_*) and disappearing during fusion (*w_Cm_*) for the PLA/PBAT matrix and biocomposites.

	Δ*h_c_* (J/g)	*w_Cc_*	Δ*h_m_* (J/g)	*w_Cm_*
base	26.0	0.26	31.0	0.26
base + 5	26.2	0.26	31.4	0.26
base + 10	25.5	0.26	32.6	0.27
base + 15	25.5	0.26	33.0	0.27
base + 20	26.2	0.26	32.8	0.27

estimated error ± 0.5 J/g for Δ*h_c_* and Δ*h_m_*, and ±0.02 for *w_Cc_* and *w_Cm._*

**Table 3:** Mechanical properties of the biocomposites.

Name	Elastic Modulus (GPa)	Maximum Strength (MPa)	Elongation at Break (%)	Charpy Impact Strength (kJ/m^2^)
base	2.26 ± 0.060	42.0 ± 0.21	312 ± 41.8	8.3 ± 0.62
base + 5	2.28 ± 0.023	40.2 ± 0.98	170 ± 36.8	4.3 ± 0.52
base + 10	2.35 ± 0.047	38.5 ± 0.50	119 ± 24.8	4.8 ± 0.57
base + 15	2.46 ± 0.119	37.2 ± 0.67	49 ± 19.9	5.0 ± 0.44
base + 20	2.62 ± 0.105	35.5 ± 1.65	7 ± 4.1	3.8 ± 1.32
